# Canine Leishmaniasis in Rio de Janeiro, Brazil (2000–2015): Taxonomic Characterisation of Etiological Agents and Geospatial Case Analysis

**DOI:** 10.1111/zph.70049

**Published:** 2026-03-11

**Authors:** Luciana de Freitas Campos Miranda, Laryssa Fabiano do Rosário Coelho, Samanta Cristina das Chagas Xavier, Artur Augusto Velho Mendes Junior, Renan Nunes Albuquerque, Aline Fagundes da Silva, Andreza Pain Marcelino, Fernanda Nunes Santos, Sandro Antonio Pereira, Rodrigo Caldas Menezes, Armando de Oliveira Schubach

**Affiliations:** ^1^ Leishmaniasis Reference Laboratory Carlos Chagas Institute, Oswaldo Cruz Foundation Fiocruz Paraná Brazil; ^2^ Laboratory of Clinical Research and Surveillance of Leishmaniasis Evandro Chagas National Institute of Infectious Diseases Fiocruz Rio de Janeiro Brazil; ^3^ Laboratory of Trypanosomatid Biology Oswaldo Cruz Institute, Oswaldo Cruz Foundation Fiocruz Rio de Janeiro Brazil; ^4^ Animal Breeding and Experimentation Laboratory Carlos Chagas Institute, Oswaldo Cruz Foundation Fiocruz Paraná Brazil; ^5^ Laboratory of Clinical Research on Dermatozoonoses in Domestic Animals Evandro Chagas National Institute of Infectious Diseases, Oswaldo Cruz Foundation Fiocruz Rio de Janeiro Brazil

**Keywords:** dogs, geographical distribution, *Leishmania braziliensis*, *Leishmania infantum*, parasitological culture, species typing

## Abstract

**Introduction:**

Canine Leishmaniasis is a vector‐borne zoonotic disease caused by several species of protozoa of the genus *Leishmania*. In the state of Rio de Janeiro (RJ), *Leishmania braziliensis* is the most prevalent species causing tegumentary leishmaniasis (TL) and *Leishmania infantum* is the main causative agent of visceral leishmaniasis (VL). Dogs are the main reservoirs of *L. infantum* and can be infected with other *Leishmania* species; however, their role as a reservoir for these species is still poorly understood. There are few epidemiological studies characterising *Leishmania* at the species level in isolates from dogs in the state of RJ and analysing their geospatial distribution.

**Methods:**

This work aimed to perform the taxonomic characterisation of *Leishmania* isolates, obtained from 565 dogs diagnosed between 2000 and 2015 in a reference centre for infectious diseases in RJ, Brazil. Dogs with a positive parasitological diagnosis by in vitro culture of different biological samples (intact skin, skin lesion, bone marrow, spleen, lymph node and others) were included. The characterisation of *Leishmania* species was carried out using multilocus enzyme electrophoresis technique. The dogs' home addresses were individually georeferenced. Thematic and heat maps were created in the QGIS software with cases and the distribution of characterised species.

**Results:**

The dogs clinically evaluated (*n* = 236) were classified as asymptomatic (*n* = 93; 39.4%), oligosymptomatic (*n* = 92; 39%), or polysymptomatic (*n* = 51; 21.6%). A total of 518 *Leishmania* isolates from dogs were characterised by MLEE as *L. infantum* (*n* = 456; 88%) and *L. braziliensis* (*n* = 62; 12%), which were obtained by cultivating biological samples from different canine sites. Heat maps identified Barra Mansa as an area of intense VL transmission and Rio de Janeiro and Maricá as municipalities with intense TL transmission.

**Conclusions:**

This study contributed to the knowledge of the taxonomic characterisation and geospatial distribution of *Leishmania* species responsible for canine leishmaniasis in the state of RJ, considering the case series from a reference centre for the diagnosis of leishmaniasis in the state of RJ, Brazil.

## Impacts

- This study identifies high‐risk areas and the main *Leishmania* species infecting dogs in Rio de Janeiro, Brazil, providing a clearer understanding of disease distribution and transmission risk.
- By confirming dogs as key hosts for parasites that also infect humans, these findings reinforce the value of canine surveillance for the early detection of zoonotic threats.
- The integration of laboratory data and geographic mapping provides actionable evidence for public health decision‐making, helping to target surveillance and control efforts more efficiently.

## 1 Introduction

Canine leishmaniasis is a vector‐borne zoonotic disease caused by protozoan parasites of the genus *Leishmania* (Kinetoplastida: Trypanosomatidae), primarily transmitted by phlebotomine sand flies (Diptera: Psychodidae: Phlebotominae). Although *Leishmania (Leishmania) infantum* is widely recognised as the main etiological agent of visceral leishmaniasis (VL) in dogs (Dantas‐Torres 2024), other species have also been associated with visceral forms of the disease. Reports of *L. (L.) amazonensis* and *L. (Viannia) lainsoni* infections in canines exhibiting clinical signs consistent with VL suggest a broader range of *Leishmania* species capable of inducing visceral pathology in this host (da Silva Santos et al. 2025; Tolezano et al. 2007). These findings underscore the need for species‐level identification in canine cases to improve diagnostic accuracy and enhance epidemiological understanding.

Domestic dogs ( *Canis lupus familiaris* ) are recognised as the primary urban reservoirs for visceral leishmaniasis (VL). The expansion of this zoonosis is a significant public health concern. Since 2006, autochthonous cases of canine visceral leishmaniasis (CVL) have been reported across various regions of the state of Rio de Janeiro (RJ), Brazil. These findings indicate a progressive geographic expansion of the disease within this specific region, underscoring the need for enhanced surveillance and control measures to mitigate its spread (Abrantes et al. 2018; de Campos et al. 2013; Madeira, de O. Schubach, et al. 2006; Madeira, Schubach, et al. 2006; de Paula et al. 2009; da Silva et al. 2011).

In Brazil, tegumentary leishmaniasis (TL) is caused by a variety of *Leishmania* species, from the two subgenera *Leishmania* and *Viannia*. The most common species are *Leishmania* (*V*.) *braziliensis*, *L*. (*V*.) *guyanensis* and *L*. (*L*.) *amazonensis*. In the Amazon region, other species can also cause TL such as *L*. (*V*.) *naiffi*, *L*. (*V*.) *lainsoni*, *L*. (*V*.) *shawi*, *L*. (*V*.) *lindenbergi* and *L*. (*V*.) *utingensis* (de Almeida et al. 2021; Lainson 2010). In RJ state, *L. braziliensis* is the most prevalent species causing TL, although autochthonous cases of *L. amazonensis* and a genetic variant of *L. naiffi* have also been described in humans (Azeredo‐Coutinho et al. 2007; Miranda, Pacheco, et al. 2019). Transmission of TL occurs in peri‐urban areas, due to disorderly human occupation (Marzochi and Marzochi 1994; Pires et al. 2014). Dogs living in areas of *L. braziliensis* transmission may present canine tegumentary leishmaniasis (CTL) characterised by cutaneous and mucosal ulcers or asymptomatic infection. Dogs can remain with active ulcers for a long time, because there is no indication for euthanasia, as occurs in CVL. However, it is unclear whether dogs participate in the transmission of *L. braziliensis* to humans (Dantas‐Torres 2007; Lago et al. 2023).

Cutaneous lesions in dogs may be caused by either *L. braziliensis* or *L. infantum*. Therefore, species‐level identification of *Leishmania* isolated from clinical samples is essential for accurate diagnosis and effective epidemiological surveillance (Madeira, de O. Schubach, et al. 2006; Madeira, Schubach, et al. 2006). Molecular or parasitological characterisation of *Leishmania* species from canine cutaneous lesions enables the differentiation between cutaneous (CTL) and visceral (CVL) leishmaniasis. This differentiation is essential for determining appropriate therapeutic strategies and, particularly in the case of CVL, for guiding disease control measures, such as euthanasia, to prevent the spread of the zoonotic infection (Ministério da Saúde [MS] 2014).

In RJ state, surveillance activities for CVL are typically triggered by municipal‐level requests following the suspicion of initial autochthonous cases. Upon such notification, environmental surveillance efforts are coordinated by the State Health Department of Rio de Janeiro (SES/RJ). These activities are conducted in collaboration with the Zoonosis Control Service and the Laboratory of Clinical Research on Dermatozoonoses in Domestic Animals (Lapclin‐Dermzoo), which is part of the Evandro Chagas National Institute of Infectious Diseases (INI)/Oswaldo Cruz Foundation (Fiocruz). During field investigations, biological samples obtained from dogs—whether collected during active surveillance or following the euthanasia of animals with laboratory‐confirmed CVL—are forwarded to the laboratory LaPClin VigiLeish/INI/Fiocruz, a reference centre for the diagnosis of leishmaniasis in RJ state.

Different biochemical and molecular methods are used to characterise *Leishmania* species. Multilocus enzyme electrophoresis (MLEE) is the reference standard technique, although laborious and applicable only to parasites that grow well in culture (Cupolillo et al. 1994; Van Der Auwera and Dujardin 2015).

Georeferencing and geoprocessing techniques are important tools that allow us to understand epidemiological and spatial patterns and thus help in surveillance and implementation of control measures (Marchi et al. 2019). In Brazil, geoprocessing in health has been used to guide actions, identify critical areas and create thematic maps, facilitating the visualisation of the distribution and incidence of events spatially (Marchi et al. 2019; Marques et al. 2017; Rodrigues et al. 2018).

Despite the endemicity of canine leishmaniasis in RJ state, there remains a paucity of studies addressing the geographic distribution of the causative *Leishmania* species, primarily due to the lack of routine taxonomic characterisation in diagnostic laboratories. This study contributes novel data to the epidemiological surveillance of canine leishmaniasis by providing, for the first time, taxonomic characterisation and georeferencing of *Leishmania* isolates obtained from dogs diagnosed at a reference centre for leishmaniasis diagnosis. The findings presented herein are of significant relevance for guiding the planning and implementation of evidence‐based strategies for the prevention and control of the disease.

## 2 Methodology

### 2.1 Study Area

**FIGURE 1:**
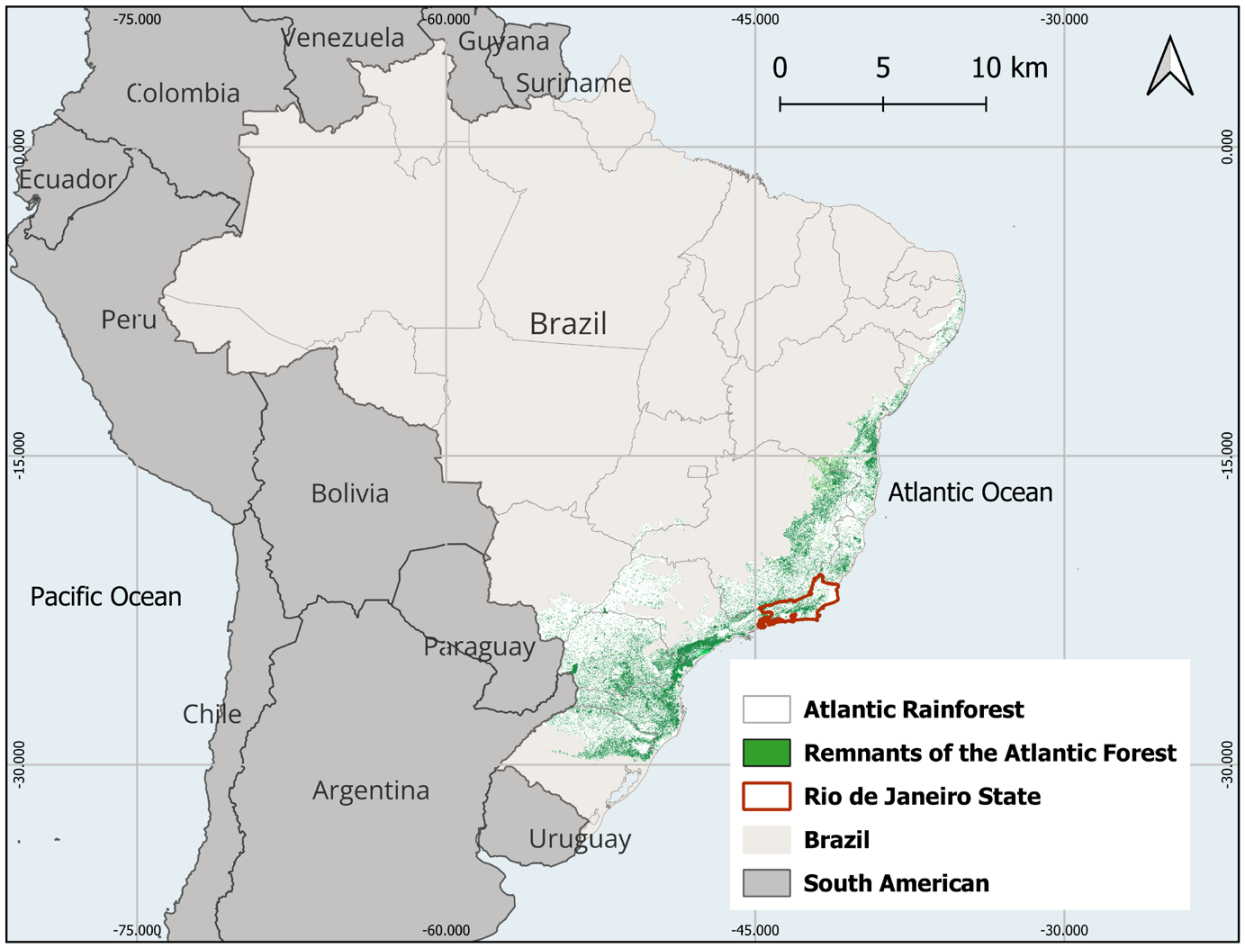
Location map of the study region: Rio de Janeiro state, Brazil, South America. This map was created using QGIS version 3.28.12 ‘Firenze’ software and cartographic bases obtained from the Brazilian Institute of Geography and Statistics (https://ibge.gov.br/).

The RJ state (22°55′S; 43°11′W) is located in the Southeast region of Brazil, within the Atlantic Rainforest biome. It has a tropical climate and comprises 92 municipalities. The estimated population is 17,219,679, spread across a territorial area of 43,750.425 km^2^, resulting in a population density of 366.97 inhabitants per km^2^ (IBGE 2024; Figure 1).

### 2.2 Cases and Samples

Retrospective study using *Leishmania* spp. isolates obtained from dogs examined at the Zoonosis Reference Center Lapclin‐Dermzoo/INI/Fiocruz, RJ‐Brazil, from 2000 to 2015. During the period of the study, 565 dogs were included, with a total of 570 biological samples. All dogs were from the RJ state, Brazil and were seen at the outpatient clinic by a veterinarian or selected in a field study after a positive serological test or the presence of compatible clinical signs of leishmaniasis. Dogs were clinically evaluated (*n* = 236) and classified as asymptomatic (no clinical signs), oligosymptomatic (1–3 signs) or polysymptomatic (4 or more clinical signs), such as thinness or cachexia; diffuse or localised alopecia; apathy; cutaneous lesions, such as ulcers and desquamation; onychogryphosis; enlargement of the superficial lymph nodes, liver or spleen on palpation; keratoconjunctivitis; pale ocular or oral mucosae, and skeletal muscle atrophy (Oliveira et al. 2021). However, the specific clinical signs could not be assessed in 329 dogs. Due to the retrospective nature of the study (2000–2015), detailed clinical records were unavailable for analysis. Dogs with a positive parasitological diagnosis by in vitro culture of at least one of the collected biological samples were included in the study. The isolated parasites kept growing until a parasitic mass was obtained, which was stored in Liquid Nitrogen (N_2_L).

### 2.3 Taxonomic Characterisation

The characterisation of *Leishmania* species was carried out from 2016 to 2022 using 518 *Leishmania* spp. isolates cryopreserved in N_2_L. To perform the parasitic mass, the isolates were grown in biphasic medium NNN (Neal, Novy, Nicolle) and Schneider's Insect Medium, added with 10% fetal bovine serum and antibiotics penicillin and streptomycin, as previously described (Miranda, da Silva, et al. 2019). The growth of the parasites was carried out in cell culture flasks until approximately 40 mL of culture was obtained, which was centrifuged to obtain the parasitic mass.

Identification of species was determined by MLEE technique, which was performed on 1% agarose gels, according to previously described procedures (Cupolillo et al. 1994). Five enzyme systems were used: 6PGDH (6‐phosphogluconate dehydrogenase, EC 1.1.1.43); GPI (glucose phosphate isomerase, EC 5.3.1.9); NH (nucleotidase, EC 3.2.2.1); G6PDH (glucose‐6‐phosphate dehydrogenase, EC 1.1.1.49) and PGM (phosphoglucomutase, EC 1.4.1.9). MLEE was performed with the reference strains of *L. braziliensis* (MHOM/BR/1975/M2903) and *L. infantum* (MHOM/BR/1974/PP75). The gel bands were analysed qualitatively, by visual comparison of the sample band profiles with the default reference strains.

### 2.4 Approach to Georeferencing and Geospatial Analysis

The geographic coordinates of the residential addresses of the dogs were obtained using the WGS‐84 Reference System (World Geodetic System—EPSG:4326). Georeferencing was done using online mapping tools: OpenStreetMap http://www.openstreetmap.org and Bing Maps http://www.bing.com/mapspreview?cc=br. To obtain better location accuracy, each address has been georeferenced individually (Ribeiro et al. 2014). In some cases it was not possible to georeference the exact addresses, so approximate addresses or centroids were used as a spatial location reference.

Thematic maps were developed in a Geographic Information System (GIS) software Quantum GIS (QGIS version 3.28.12 ‘Firenze’; https://www.qgis.org). The spatial analysis utilised cartographic bases of the RJ state from Brazilian Institute of Geography and Statistics (IBGE).

#### 2.4.1 Class Maps

Represent the number of cases of canine leishmaniasis diagnosed from 2000 to 2015 in the LaPClin VigiLeish reference centre. The classes were defined by the equal interval method. The colour gradations of the classes were represented with white representing municipalities with no registered cases and the grey to black scale for the intervals of 1–288 canine cases.

#### 2.4.2 Point Maps

Represent the *Leishmania* species taxonomically characterised according to the dogs' residential addresses. Specific colours and symbols were used to identify each *Leishmania* species.

#### 2.4.3 Heat Maps (Kernel Density)

Represent the areas of mild, moderate or intense transmission of canine leishmaniasis in RJ state, considering all diagnosed cases. The Kernel Function (k) adopted was Quartic, which gives greater weight to the closest points than to distant points, but the decrease is gradual and the size of the radius was defined through the distance matrix (*R* = $\bar{X}$ ± $\bar{X}$σ) for each type of *Leishmania* species (Rizzatti et al. 2020).

## 3 Results

### 3.1 Clinical Evaluation of Dogs

Of the 565 dogs included in the study, 236 (41.8%) were clinically evaluated by Lapclin‐Dermzoo veterinarians and classified as asymptomatic (*n* = 93; 39.4%), oligosymptomatic (*n* = 92; 39%), or polysymptomatic (*n* = 51; 21.6%).

The main clinical signs were weight loss (*n* = 63; 26.7%), onychogryphosis (*n* = 62; 26.3%), skin scaling (*n* = 57; 24.1%), lymphadenomegaly (*n* = 55; 23.3%), splenomegaly (*n* = 50; 21.2%), ophthalmic changes (*n* = 49; 20.7%), alopecia (*n* = 37; 15.7%), cachexia (*n* = 26; 11%), crusted ulcer (*n* = 26; 11%) and hepatomegaly (*n* = 10; 4.2%).

### 3.2 Canine Samples and Taxonomic Characterisation

*Leishmania* spp. isolates were lost due to contamination or lack of parasite growth after cryopreservation (*n* = 52). A total of 518 *Leishmania* isolates from dogs were characterised by MLEE as *L. infantum* (*n* = 456; 88%) and *L. braziliensis* (*n* = 62; 12%), which were obtained by cultivating biological samples from different canine sites. Samples were obtained from various sites, with intact skin being the most frequent source. The spleen was the second most common site, followed by bone marrow aspirates and skin lesions (Table 1).

**TABLE 1:** Distribution of *Leishmania* species according to sites of parasite isolation in dogs from Rio de Janeiro State, Brazil, 2000–2015 (*N*, %).

Site of isolation *N* (%)	*Leishmania infantum N* (%)	*Leishmania braziliensis N* (%)	Total number of species characterised *N* (%)
Intact skin (*N* = 159; 27.9)	140 (88)	2 (1.3)	142 (89.3)
Spleen (*N* = 143; 25.1)	127 (88.8)	0 (0)	127 (88.8)
Bone marrow aspirate (*N* = 91; 16)	85 (93.4)	1 (1.1)	86 (94.5)
Skin lesion (*N* = 75; 13.1)	17 (22.7)	56 (74.7)	73 (97.4)
Lymph node fragment (*N* = 73; 12.8)	65 (89)	0 (0)	65 (89)
Lymph node aspirate (*N* = 8; 1.4)	7 (87.5)	0 (0)	7 (87.5)
Cerebrospinal fluid (*N* = 3; 0.5)	3 (100)	0 (0)	3 (100)
Nasal mucosa lesion (*N* = 2; 0.35)	0 (0)	2 (100)	2 (100)
Blood (*N* = 2; 0.35)	2 (100)	0 (0)	2 (100)
Liver (*N* = 2; 0.35)	2 (100)	0 (0)	2 (100)
Thymus (*N* = 2; 0.35)	2 (100)	0 (0)	2 (100)
Skin scar (*N* = 1; 0.2)	0 (0)	1 (100)	1 (100)
Vulva (*N* = 1; 0.2)	1 (100)	0 (0)	1 (100)
Uterus (*N* = 1; 0.2)	1 (100)	0 (0)	1 (100)
Ignored (*N* = 7; 1.2)	4 (100)	0 (0)	4 (100)
TOTAL OF SAMPLES *N* = 570 (100)	456 (80)	62 (10.9)	518 (90.9)

Notably, *L. braziliensis* was predominantly isolated from skin lesions (*n* = 56), while *L. infantum* was found in only 17 of these samples, suggesting a possible association of *L. braziliensis* with localised cutaneous manifestations. All isolates from lymph node aspirates and fragments were characterised as *L. infantum*. Other less frequent isolation sites included lymph node aspirates, cerebrospinal fluid, blood, liver, thymus, skin scars, vulva and uterus. In 1.2% of samples, the isolation site was not specified (Table 1).

Mixed infections with *L. infantum* and *L. braziliensis* were identified in five dogs (0.88%), with isolates obtained from different anatomical sites. In most cases, *L. infantum* was found in internal organs or intact skin, while *L. braziliensis* was associated with cutaneous or mucosal lesions, suggesting distinct tissue tropism between the two species (Table 2).

**TABLE 2:** Isolation sites from dogs with mixed infection by *Leishmania braziliensis* and *Leishmania infantum*.

Dog with mixed Infection	Isolation site of *L. infantum*	Isolation site of *L. braziliensis*
D1	Ignored	Skin lesion
D2	Lymph node	Skin lesion
D3	Ignored	Skin scar
D4	Intact skin	Nasal mucosa lesion
D5	Spleen	Skin lesion

*Note:* Dogs domiciled in the municipality of Rio de Janeiro were evaluated at the reference centre for leishmaniasis between 2000 and 2015.

### 3.3 Georeferencing and Geospatial Analysis

The addresses of the 565 dogs were obtained from the medical records of these animals, and the geographic coordinates were obtained through the online cartographic databases Bing Maps (*n* = 330; 58.5%) or OpenStreetMap (*n* = 235; 41.5%). Subsequently, these georeferenced data were used to create thematic maps with the distribution of cases with the respective characterised species. Georeferencing was performed using the exact address (*n* = 298; 52.74%), neighbourhood centroid (*n* = 194; 34.33%), approximate address (*n* = 37; 6.54%) or centroid of the municipality (*n* = 36; 6.37%).

The analysis of the geographic distribution of canine samples revealed that 23/92 municipalities in RJ state presented cases of canine leishmaniasis. Of these, 11 municipalities presented only cases of infection by *L. infantum*, three presented only cases of infection by *L. braziliensis* , and eight municipalities presented cases of infection by both species. The municipality of Barra do Piraí was the only one with a case of CVL whose species of *Leishmania* was not identified. The municipality with the highest number of records was Barra Mansa (*n* = 288), where *L. infantum* predominated (*n* = 261). The capital, Rio de Janeiro, had the second highest number of cases (*n* = 196), with a higher proportion of *L. infantum* (*n* = 146) compared to *L. braziliensis* (*n* = 30). The municipalities of Maricá, where *L. braziliensis* accounted for the majority of cases (*n* = 19/29) and Mangaratiba, which had more cases of *L. infantum* (*n* = 9/13), were the third and fourth with the highest number of canine cases, respectively (Table 3).

**TABLE 3:** Geographic distribution and number of cases of canine infection by *Leishmania* species by municipality in Rio de Janeiro state, Brazil (2000–2015).

Municipality	*Leishmania infantum*	*Leishmania braziliensis*	*Leishmania* spp.^a^	Total
ANGRA DOS REIS	1	1	0	2
ARARUAMA	1	0	0	1
BARRA DO PIRAÍ	0	0	1	1
BARRA MANSA	261	2	25	288
BELFORD ROXO	1	0	0	1
CAMPOS DOS GOYTACAZES	1	0	0	1
ITAPERUNA	1	0	0	1
ITATIAIA	1	0	0	1
MANGARATIBA	9	1	3	13
MARICÁ	10	19	0	29
MIGUEL PEREIRA	0	1	0	1
NITERÓI	7	1	0	8
NOVA FRIBURGO	1	0	0	1
PARACAMBI	0	1	1	2
PARATY	6	0	0	6
PETRÓPOLIS	0	1	1	2
PIRAÍ	1	3	0	4
QUEIMADOS	1	0	0	1
RESENDE	2	0	0	2
RIO DE JANEIRO^b^	146	30	20	196
SEROPÉDICA	4	2	1	7
VASSOURAS	1	0	0	1
VOLTA REDONDA	1	0	0	1
TOTAL	456	62	52	570

^a^ = *Leishmania* spp. = isolates identified as *Leishmania* but not characterized at the species level.

^b^ = Rio de Janeiro municipality (capital of the state of Rio de Janeiro).

**FIGURE 2:**
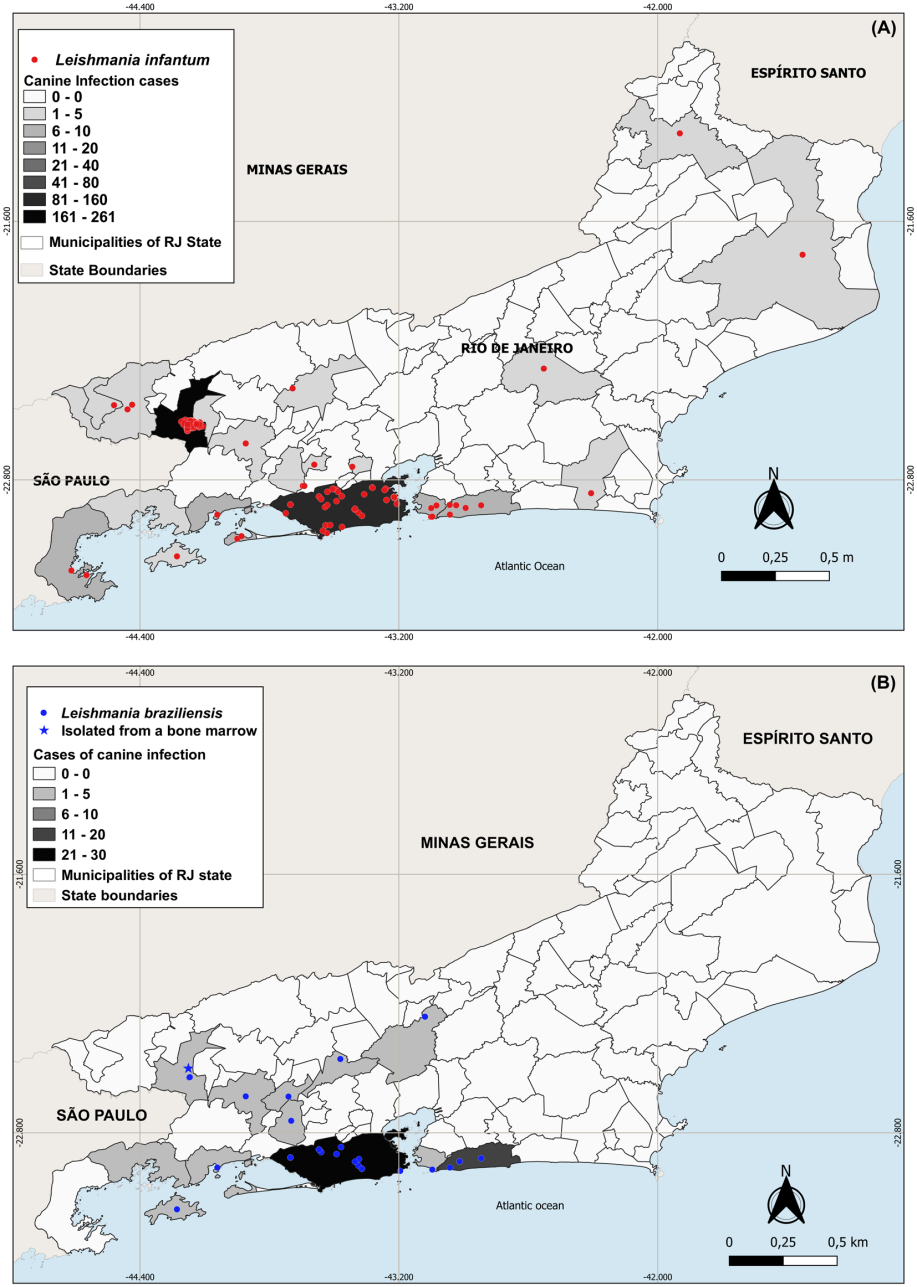
(A, B). Geospatial distribution patterns of *Leishmania* species were analysed using thematic cartographic methods. (A) shows a classified map of canine infection cases by *L. infantum*, while (B) displays a classified map of canine infection cases by *L. braziliensis* , based on diagnoses from dogs evaluated at the reference centre for leishmaniasis between 2000 and 2015. Case classes were defined using the equal interval classification method. Maps were created in QGIS 3.28.12 ‘Firenze’, using as a cartographic base the state map of Rio de Janeiro provided by the Brazilian Institute of Geography and Statistics (https://ibge.gov.br).

Overall, *L. infantum* exhibited a broader and more intense spatial distribution compared to *L. braziliensis* . The highest concentration of *L. infantum* cases was recorded in the municipality of Rio de Janeiro and in the southern region of the state, particularly in the municipality of Barra Mansa, with 261 CVL cases reported. In contrast, other municipalities, especially those in the central‐southern and coastal regions, presented a scattered distribution pattern, with most reporting between 1 and 20 cases. The spatial distribution of *L. braziliensis* was more limited and localised, with the majority of cases concentrated in the metropolitan area and in southern municipalities of the state, where the number of cases ranged from 11 to 30 (Figure 2A,B).

**FIGURE 3:**
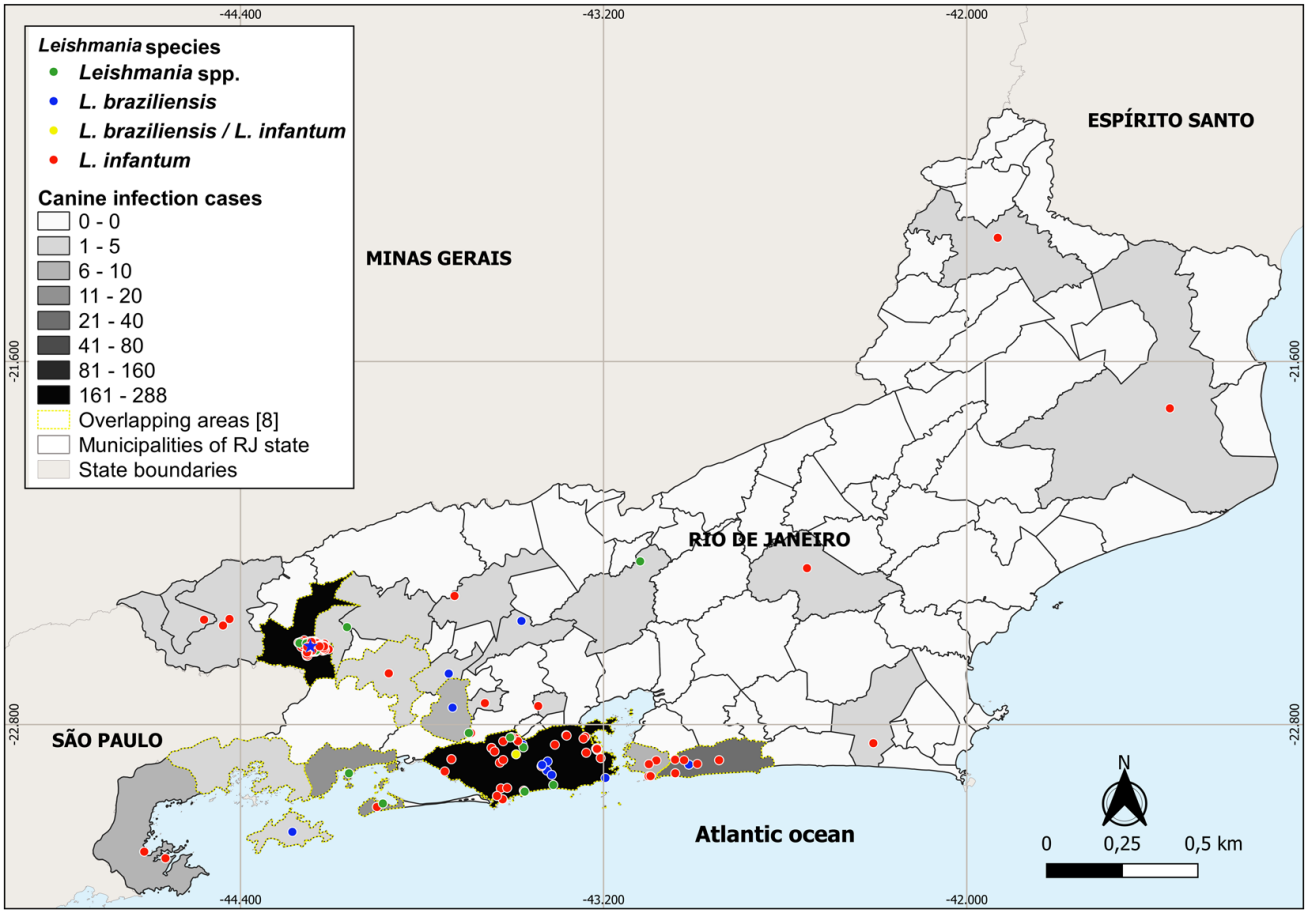
Classified map of the total number of canine leishmaniasis cases, showing the locations of all *Leishmania* species identified in dogs from the Rio de Janeiro state, Brazil, diagnosed at the leishmaniasis reference centre between 2000 and 2015. Case classes were defined using the equal interval classification method. The map was created in QGIS 3.28.12 ‘Firenze’ using the state map of Rio de Janeiro provided by the Brazilian Institute of Geography and Statistics (https://ibge.gov.br) as the cartographic base.

The map reveals a clear hotspot of canine leishmaniasis in the densely populated southeastern region of Rio de Janeiro, especially along the southern coast and around the capital, where case numbers reached the highest levels. *L. infantum* was the most widespread species, while *L. braziliensis* showed a more localised presence. Co‐infections and unidentified species were less frequent and concentrated in high‐burden areas. The areas highlighted in yellow on the map indicate municipalities with spatial overlap of canine infection transmission of *L. infantum* and *L. braziliensis* (Figure 3).

**FIGURE 4:**
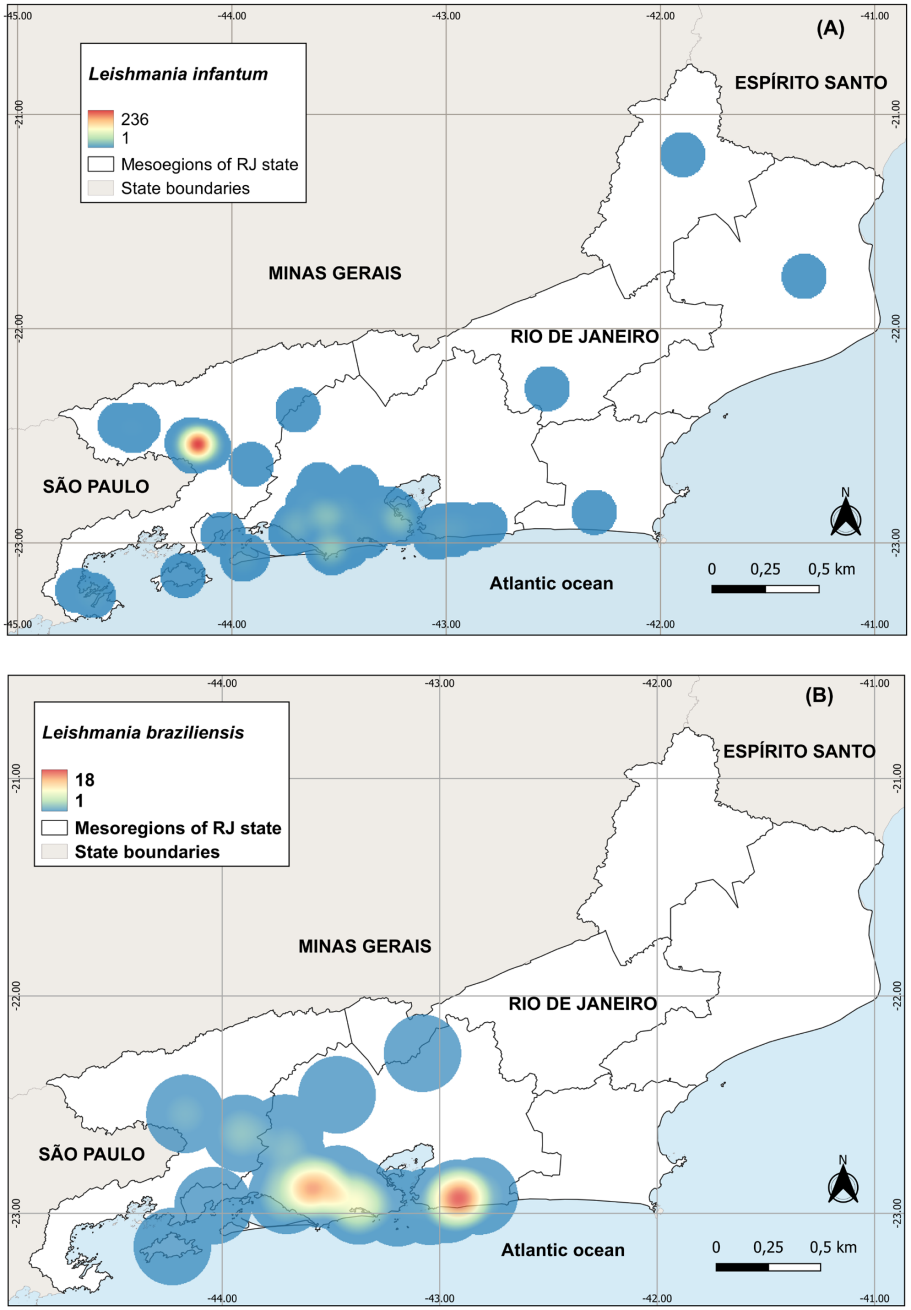
(A, B) Mapping the spatial distribution of canine leishmaniasis in the state of Rio de Janeiro from 2000 to 2015: The heat maps illustrate the total number of cases for CVL (A) and CTL (B), highlighting regions with varying case densities: Low, medium‐low, medium, medium‐high and high. The map was created using QGIS 3.28.12 ‘Firenze’ software, with the cartographic base sourced from the Brazilian Institute of Geography and Statistics (https://ibge.gov.br).

Figure 4A,B presents kernel density heat maps generated from the total number of canine leishmaniasis cases, highlighting areas of low, medium and high case density across the RJ state. These spatial patterns underscore priority regions for targeted surveillance and control efforts, reflecting species‐specific transmission dynamics. The distribution of *L. infantum* (CVL) reveals a pronounced hotspot in the metropolitan area, particularly in municipalities of the Baixada Fluminense, indicating a zone of intense transmission. Additional clusters of moderate density are observed in the southern and southeastern regions of the state. In contrast, the distribution of *L. braziliensis* (CTL) is more localised, with distinct density clusters concentrated in the southern region, especially in coastal municipalities.

## 4 Discussion

Dogs play an important role in the cycle of VL caused by *L. infantum*, mainly because they present the infective forms of this parasite species on their intact skin, becoming a source of infection for the insect vector, increasing the risk of transmission to humans. In this study, 30.7% (*n* = 140) of the species characterised as *L. infantum* were isolated from the intact skin of dogs, reinforcing the potential of dogs as urban reservoirs of the disease. However, the role of dogs as reservoirs of TL has not yet been established for dogs infected by dermotropic species of *Leishmania* (Dantas‐Torres 2007; Lago et al. 2023). Although euthanasia is indicated exclusively for dogs infected with *L. infantum*, dogs infected with other species of *Leishmania* spp. and with reactive serological tests for CVL are subjected to euthanasia (Grimaldi et al. 2012). Despite the relevance of species identification in clinical and epidemiological aspects, this is not routinely performed in most laboratories, and is generally limited to specialised laboratories in research centres (Schwarz et al. 2017).

Among the 565 animals included in this study, 236 underwent clinical evaluation. The majority of these dogs (*n* = 143; 60.6%) exhibited clinical signs suggestive of leishmaniasis. Notably, weight loss was the most prevalent finding, which diverged from the literature where dermatological manifestations are typically reported as the most frequent (Saridomichelakis et al. 2025). In contrast to studies where asymptomatic dogs predominated (Alvar et al. 2004; Dantas‐Torres et al. 2006), our results were likely influenced by the inclusion criteria, which targeted animals with either clinical suspicion or seropositivity. This approach may have led to an overrepresentation of clinically affected animals. Furthermore, the retrospective nature of the study (2000–2015) resulted in missing clinical information for 329 animals. Despite this limitation, the remaining sample provided sufficient power to characterise the predominant clinical manifestations in the region.

In the present study, all cases of CTL were caused by *L. braziliensis* . Considering that most human cases of TL in RJ state are caused by this species, our findings corroborate the local epidemiological pattern (Miranda, Pacheco, et al. 2019). Furthermore, this result highlights the importance of expanding studies on the relationship between dogs and other animals as possible reservoirs of TL in the urban environment (Lago et al. 2023).

The same species of *Leishmania* can generate different phenotypes of the disease, thus varying its tropism and clinical manifestations (Silva et al. 2021). There are reports of *L. amazonensis* causing CVL (Dias et al. 2011; Tolezano et al. 2007; Valdivia et al. 2017). However, this study did not identify *L. amazonensis* in any case, a fact that may be linked to the low circulation of this species in RJ state (Azeredo‐Coutinho et al. 2007).

A dog from the municipality of Barra Mansa had *Leishmania* isolated from a bone marrow sample, which was identified as *L. braziliensis* by MLEE. This dermotropic species is usually isolated from skin lesions or scars. Recently, there was an unprecedented report of visceralization of *L. lainsoni* in a dog living in the same municipality (Santos et al. 2025). Visceralization of *L. braziliensis* has been documented in immunocompromised human patients (Gontijo et al. 2002; da Silva et al. 2002). In contrast, the absence of similar reports in dogs highlights this discovery as an unprecedented event with considerable epidemiological importance.

Furthermore, we identified five dogs with mixed infections by *L. infantum* and *L. braziliensis* , confirming literature reports of mixed infections detected in canine cases in the Rio de Janeiro municipality (Madeira, Schubach, et al. 2006; Pires et al. 2014).

Heat maps allowed us to identify the areas with the highest density of cases (hot spots) of canine leishmaniasis in RJ state. This information is important to guide surveillance actions in the studied areas. The municipality of Barra Mansa had the highest intensity of CVL transmission in the RJ state. In this municipality, there was a survey between 2011 and 2013 to identify *Leishmania* species in seroreactive dogs, identifying *L. infantum* as the etiological agent in this area (de Mello et al. 2014). Although some surveillance actions to control CVL have been carried out, new canine cases have been reported in several municipalities in the RJ state, with notifications in cities such as Mangaratiba, Maricá, Niterói, Barra Mansa, Cachoeiras de Macacú, Volta Redonda, Resende and Rio de Janeiro (da Silva et al. 2015). In 2009, the first autochthonous case of CVL was recorded in the Jacaré neighbourhood, in the municipality of Niterói, triggering an epidemiological investigation in the area from 2010 to 2011, with detection of seropositive dogs in the immunoenzymatic assay (ELISA), Dual Path Platform and indirect immunofluorescence tests (de Oliveira et al. 2015).

The municipalities of Maricá and Rio de Janeiro were identified as hot spots for CTL transmission, confirming previous reports that these municipalities have cases of both CTL and CVL (Abrantes et al. 2016; Abrantes et al. 2018; Antunes Uchôa et al. 2001; Madeira, de O. Schubach, et al. 2006; de Oliveira et al. 2015; de Paula et al. 2009; Serra et al. 2003; da Silva et al. 2015). An analysis of the spatial distribution of cutaneous leishmaniasis in the municipality of Rio de Janeiro reveals a higher concentration of cases in neighbourhoods located near mountainous regions, particularly in areas adjacent to the Pedra Branca Massif and the Tijuca Forest. This spatial correlation suggests an epidemiological link between CL occurrence and environmental characteristics associated with these peri‐mountainous ecosystems (Kawa and Sabroza 2002). Species of phlebotomine sandflies, including *Nyssomyia intermedia*, *Nyssomyia whitmani*, *Migonemyia migonei*, and members of the *Psychodopygus wellcomei* complex, have been reported as competent vectors of *Leishmania* spp. associated with the transmission of cutaneous leishmaniasis in the municipality of Rio de Janeiro (Carvalho et al. 2014).

An entomological survey was carried out in the Serra da Tiririca State Park, a legally protected Atlantic Forest remnant located in a mountainous area bordering the municipalities of Maricá and Niterói, in the metropolitan region of Rio de Janeiro state, Brazil. A total of 12 phlebotomine sand fly species were recorded. *Migonemyia migonei* and *Nyssomyia intermedia* were identified as putative vectors of cutaneous leishmaniasis in the area (Rodrigues et al. 2013).

According to the known epidemiological dynamics of leishmaniasis transmission, canine cases often precede the occurrence of human cases, serving as sentinels for human infection risk (Ministério da Saúde and Brasil 2014; Teixeira‐Neto et al. 2014). In the present study, this pattern was corroborated by the temporal and spatial association observed between confirmed cases of CVL in the municipality of Barra Mansa and subsequent human cases reported in the same region, including instances that resulted in fatal outcomes (de Mello et al. 2014; Pimentel et al. 2014). Regarding tegumentary leishmaniasis, the municipality of Rio de Janeiro exhibits a high incidence of both human and canine cases, indicating a potential overlap in transmission cycles (Madeira, de O. Schubach, et al. 2006; Madeira, Schubach, et al. 2006; Miranda, Pacheco, et al. 2019). In contrast, no such association was identified in the municipality of Maricá, where only canine infections were detected and no autochthonous human cases have been reported to date.

Although Multilocus Enzyme Electrophoresis (MLEE) remains a robust reference technique for the characterisation of *Leishmania* isolates, as demonstrated by the successful identification of 518 samples in this study, we acknowledge the potential benefits of integrating molecular approaches in future research. The adoption of PCR‐based methods alongside MLEE could serve as a valuable complement, particularly in clinical scenarios where the parasite burden is low or where successful parasite culture is not feasible. Such molecular tools would not only enhance species detection sensitivity but also facilitate broader comparisons across different epidemiological studies, providing a more comprehensive understanding of *Leishmania* distribution across diverse anatomical sites (Akhoundi et al. 2017; Schönian et al. 2011).

## 5 Conclusion

This study analysed a 15‐year canine case series (2000–2015) from a diagnostic reference centre in leishmaniasis and analysed the taxonomic characterisation and spatial distribution of *Leishmania* species associated with canine leishmaniasis in RJ state, Brazil. The findings provide essential epidemiological insights that support the development of targeted surveillance and control strategies, highlighting the need for ongoing molecular and epidemiological monitoring to detect changes in species distribution and guide public health interventions.

## Funding

This study was funded by grants from Coordenação de Aperfeiçoamento de Pessoal de Nível Superior‐CAPES Brazil (LFRC, Finance code: 001), Fundação Carlos Chagas de Amparo à Pesquisa do Estado do Rio de Janeiro (FAPERJ) E‐26/202.911/2015, E‐26/202.737/2019, E‐26/200.791/2020, E‐26/201.032/2021, E‐26/201.314/2021 and E‐26/210.063/2024. Conselho Nacional de Desenvolvimento Científico e Tecnológico (CNPq), Brazil (Grants 304.335/2014‐2, 302.414/2018‐5, 309.657/2016‐4, 305.956/2021‐3 and 316.975/2023‐0).

## Ethics Statement

The study protocol was approved by the Ethics Committee on Animal Use of the Oswaldo Cruz Foundation (CEUA/Fiocruz) with Licence Numbers: P195‐03, L‐038/08 and LW‐54/13.

## Conflicts of Interest

The authors declare no conflicts of interest.

## Data Availability

The data supporting the findings of this study are included within the article. Additional raw data or specific datasets are available from the corresponding author upon reasonable request.
